# Mechanotransduction in distinct F-actin architectures: a novel molecular tension sensor revealing cellular mechanical anisotropy

**DOI:** 10.1016/j.mbm.2024.100045

**Published:** 2024-02-06

**Authors:** Ting Liang, Bin Li

**Affiliations:** Medical 3D Printing Center, Orthopedic Institute, Department of Orthopedic Surgery, The First Affiliated Hospital, School of Biology and Basic Medical Sciences, Suzhou Medical College, Soochow University, Suzhou, Jiangsu 215000, PR China

**Keywords:** Mechanotransducton, F-actin, Tension sensor, Mechanical anisotropy, Traction force microscopy

## Abstract

Mechanotransduction is essential for cell fate and behavior, and F-actin plays a key role in the generation and transmission of molecular forces. A recent study published in *Nature Communication* presented a novel high-precision molecular tension measurement method using a Förster resonance energy transfer–based tension sensor with separated load-bearing function within distinct F-actin structures, and demonstrated that cellular mechanical anisotropy depends on cell shape, loading direction, and magnitude.

The mechanical environment of the extracellular matrix (ECM), including the matrix mechanical properties (stiffness or viscoelasticity) and dynamic loading, regulates cell fate and behavior via cell-ECM mechanotransduction [1,2]. During this process, F-actin senses and responds to external mechanical cues and plays a key role in the generation and transmission of cell-ECM forces [3,4]. The actin network also determines cell stiffness and influences cell differentiation through integrin [5] and subsequently, Yes-associated protein (YAP) [6,7]. However, the detailed mechanisms regulating the production and transmission of local forces through F-actin remain unclear.

The F-actin cytoskeleton is composed of stress fibers and cortical filaments that form a mechanical continuum and produce contractile force [8,9]. Although stress fibers and cortical actin both contract via myosin motors, they differ in their nucleation and organization [10,11]. Under mechanical loading, how these different cytoskeletons work and how they coordinate are essential for determining cell shape, mechanotransduction, and stress–strain constitutive relationships, which remain to be defined.

A recent study reported a novel, high-precision molecular tension measurement method using a Förster resonance energy transfer (FRET)–based tension sensor [12] (Fig. 1). The sensor was embedded in F-actin assemblies and did not perturb the cytoskeleton. By quantifying the different densities of the sensor in different F-actin structures, this tension sensor was enriched within different structures to different extents and further showed differences in the degree of localization between stress fibers and cortical actin. The authors then correlated the molecular tension within F-actin with the traction forces by comparing the FRET efficiency within cells to the traction vectors measured by traction force microscopy (TFM) after activating and inhibiting myosin activity. After this correlation, the tension sensor could report the molecular tension within the stress fibers and cortical actin.

**Fig. 1:**
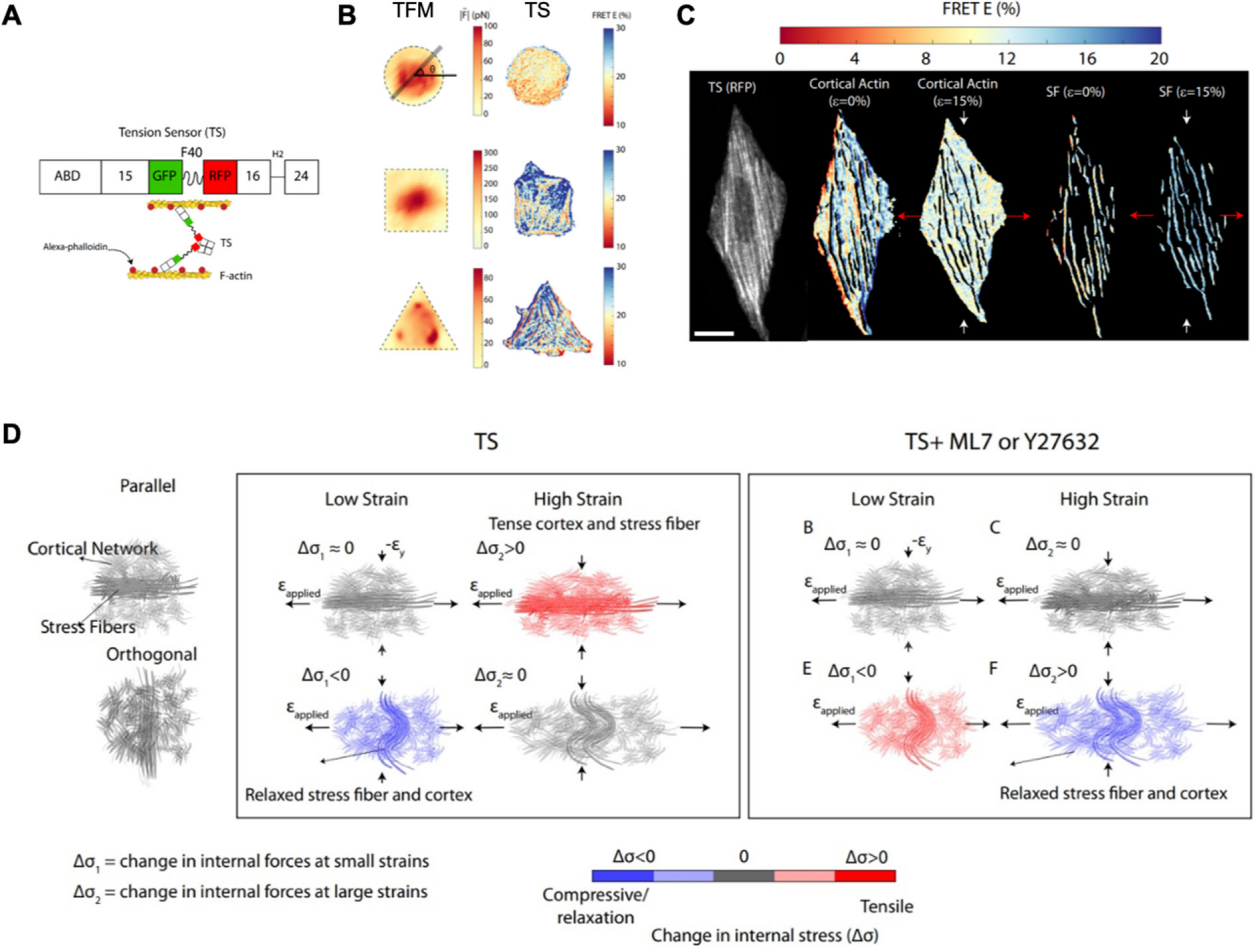
The role of distinct F-actin structures and myosin activity in mechanical anisotropy of the cytoskeleton. (A) A schematic of the tension sensor (TS). (B) Differences in the intracellular force distribution between traction force microscopy (TFM; elastic assumption) and tension sensor (TS) techniques within circular, square, and triangular cells. (C) Difference in load bearing between stress fibers and cortical actin under tensile strain of 0 and 15%, respectively. (D) Integrated stress fiber and cortical network under low and high applied strains parallel to the direction of the stress fiber, and under myosin inhibitor ML7 or Y27632 treatment [12].

Next, the authors established cells with circular, square, and triangular shapes and compared the internal forces (|$\tilde{F}$|) obtained by TFM based on homogenous-isotropic elasticity to the distribution of FRET. |$\tilde{F}$| exhibited a homogeneous distribution similar to that of FRET for circular cells, whereas the stress fibers were distributed across the central area nearly uniformly. For the square and triangular cells, |$\tilde{F}$| exhibited a heterogeneous distribution different from that of FRET, whereas the stress fibers were predominantly peripheral and presented perpendicular or angled arrangements. Thus, these differences between FRET and |$\tilde{F}$| indicate the limitation of the assumption of homogeneous-isotropic elasticity of cells owing to the cytoskeletal organization of different cell shapes.

To further explore the effect of stress fiber alignment on mechanical anisotropy, the authors controlled the stress fiber orientation using grooved polydimethylsiloxane (PDMS) films and loaded uniaxial stretching on cells with parallel or orthogonal alignment. A tensile strain of 5% did not change FRET significantly for parallel cells, but induced a positive change in FRET in orthogonal cells. At higher strains of 15% and 20%, the change in FRET decreased in parallel cells but did not change significantly in orthogonal cells. Because the change in FRET depended on the stretching direction, the cell stress–strain response exhibited mechanical anisotropy. After myosin inhibition, the change in FRET was absent or attenuated at higher strains, but showed a tensile response at 5% strain. When branched actin in the cortex was disrupted, changes in FRET also differed between parallel and orthogonal cells under different strains. These results indicate that the mechanical anisotropy in the cells is strain-dependent.

Finally, F-actin masks were generated to separate the different regions corresponding to stress fibers and cortical action in the fluorescence images. During cell stretching, the change in the FRET of stress fibers and cortical actin decreased in parallel cells. In contrast, in orthogonal cells, changes in stress fibers increased, and cortical actin levels decreased. After myosin inhibition, cortical actin generated more molecular forces than stress fibers. These results indicate that the mechanical anisotropy of cells depends on the strain direction and different load bearings between the stress fibers and cortical actin.

In conclusion, this study presents a novel FRET sensor for measuring the molecular tension in cells while separating stress fibers and cortical actin. The different changes in FRET showed dependence on the cell shape, strain magnitude, and loading direction, which was concluded to be mechanical anisotropy. The difference in load bearing between stress fibers and cortical actin was also revealed, which provided a better understanding of the generation and transmission of molecular forces.

## Ethical approval

This study does not contain any studies with human or animal subjects performed by any of the authors.

## Declaration of competing interest

The authors declare that they have no known competing financial interests or personal relationships that could have appeared to influence the work reported in this paper.
